# Supplementary material for: Differential splice isoforms of mouse CDK2 play functionally redundant roles during mitotic and meiotic division
Source: J Cell Sci. 2025 Oct 10;138(19):jcs264291. doi: 10.1242/jcs.264291 (PMC12539205; doi:10.1242/jcs.264291)
Supplement: Supplementary information [file joces-138-264291-s1.pdf]

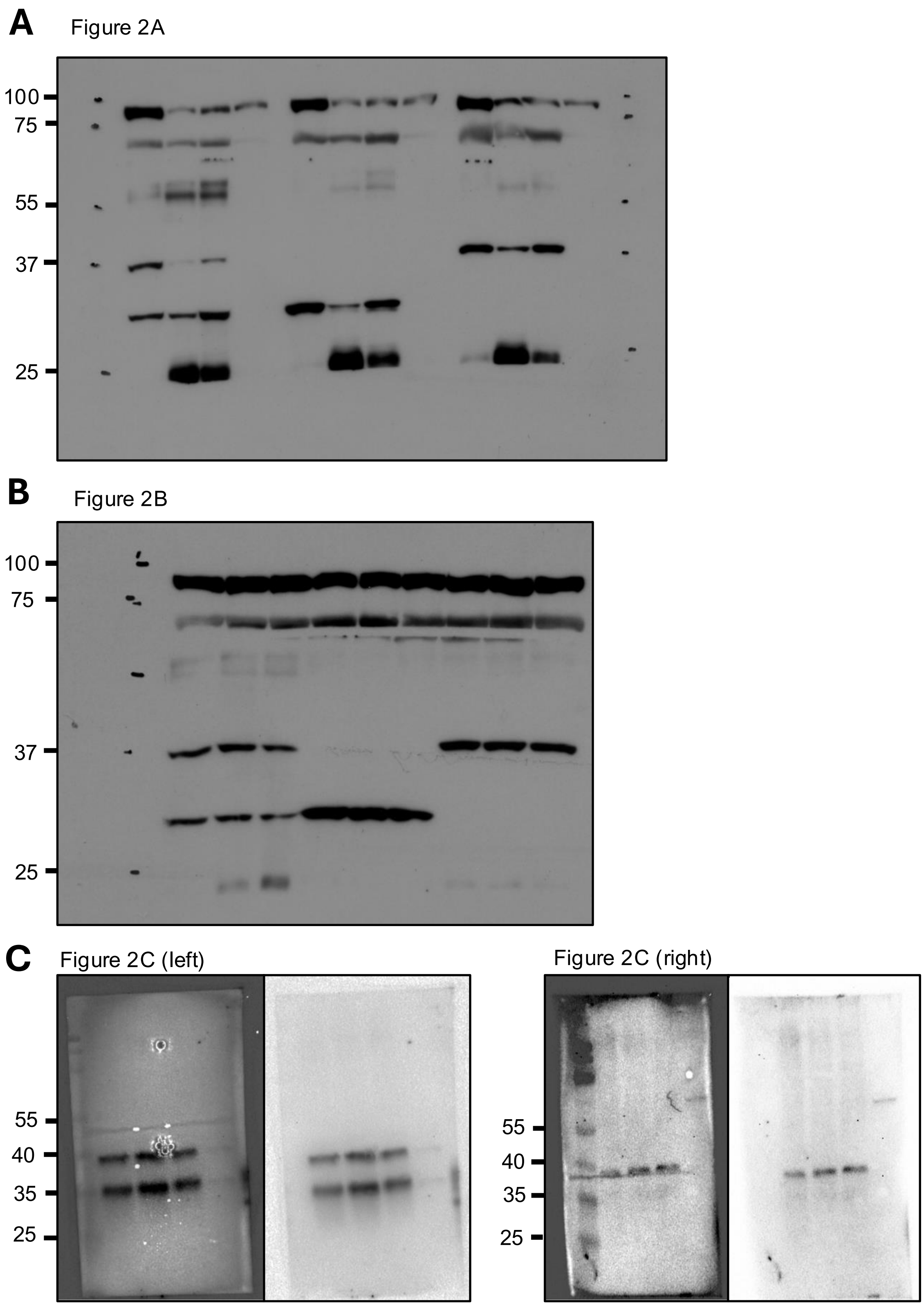

Fig. S1. Blot transparency. Uncropped Western blots from Fig. 2A (A) Cdk2 in Testis, Spleen, Thymus, Brain tissues of wild type, Cdk2$^{SHORT/SHORT}$, Cdk2$^{LONG/LONG}$ animals, and loading control HSP90, (B) from Figure 2B Cdk2 in testis, in wildtype wild type, Cdk2$^{SHORT/SHORT}$, Cdk2$^{LONG/LONG}$ animals, and loading control HSP90, (C) from Fig. 2C, Validation of antibody against Cdk2L.

Journal of Cell Science • Supplementary information
